# Circulating Adipokine Levels in Nonobese Women With Polycystic Ovary Syndrome and in Nonobese Control Women: A Systematic Review and Meta-Analysis

**DOI:** 10.3389/fendo.2020.537809

**Published:** 2021-01-07

**Authors:** Kainan Lin, Xiaoting Sun, Xiao Wang, Hanchu Wang, Xia Chen

**Affiliations:** ^1^ Reproductive Medicine Center, The First Afﬁliated Hospital of Wenzhou Medical University, Wenzhou, China; ^2^ Department of Obstetrics and Gynecology, The First Affiliated Hospital of Wenzhou Medical University, Wenzhou, China

**Keywords:** polycystic ovary syndrome, adipokine, systematic review, meta-analysis, non-obese

## Abstract

Levels of circulating adipokines in nonobese polycystic ovary syndrome (PCOS) patients have been reported in many studies. However, the results are inconsistent. The aim of this meta-analysis is to assess whether the levels of circulating adipokines are changed in nonobese PCOS relative to nonobese healthy controls. To identify eligible studies, a literature research was performed in the PubMed, Embase, and Web of Science databases without restricting by region, journal, or language. A total of 81 studies met the eligibility criteria. The meta-analysis showed that the circulating level of adiponectin (standardized mean difference [SMD]: -0.95; 95% CI: -1.36 to -0.53) was significantly decreased in nonobese PCOS patients. In contrast, the circulating levels of chemerin (SMD: 1.13; 95% CI: 0.08 to 2.18), leptin (SMD: 0.47; 95% CI: 0.13 to 0.81), resistin (SMD: 0.45; 95% CI: 0.03 to 0.88), and visfatin (SMD: 1.38; 95% CI: 0.68 to 2.09) were significantly increased in nonobese PCOS patients. There were no significant changes in the circulating levels of apelin (SMD: 0.32; 95% CI: -1.34 to 1.99), irisin (SMD: 1.01; 95% CI: -0.68 to 2.70), omentin (SMD: -0.37; 95% CI: -1.05 to 0.31), or vaspin (SMD: 0.09; 95% CI: -0.14 to 0.32). Thus, scientific evidence suggests that the circulating adipokine levels are altered in nonobese PCOS patients compared to nonobese healthy controls. Therefore, independent of the degree of obesity, dysregulated circulating adipokine levels might play important roles in the occurrence and development of PCOS.

## Introduction

Polycystic ovary syndrome (PCOS) is a common endocrine and metabolic disease. It affects 6%–10% of reproductive-aged women, according to various diagnostic criteria (1). It is diagnosed based on sparse ovulation or anovulation, hyperandrogenism, and/or polycystic ovaries (2). Research has shown that the obesity rate among PCOS patients is significantly increased, which suggests that obesity may be related to the occurrence and symptoms of PCOS (3).

Fat tissue, which we often think of as a storage site for energy, is a crucial endocrine tissue in the body (4). Adipokines are active hormones and other factors that are secreted by adipocytes. Examples include adiponectin, apelin, chemerin, irisin, leptin, omentin, resistin, vaspin, and visfatin. As adipokines are involved in many critical physiological processes in the body, dysregulation of adipokines may lead to endocrine diseases (5). Additionally, research has shown that adipokines play an essential role in the pathogenesis of obesity and obesity-related diseases (6).

PCOS patients commonly have metabolic complications, such as type 2 diabetes, insulin resistance (IR), and adipose tissue dysfunction (7–9). IR affects about 10%–25% of the general population, but PCOS patients have 2–3 times the risk of IR (10, 11). In addition, the incidence of obesity is higher in patients with PCOS (relative risk [RR]: 2.77; 95% CI: 1.88 to 4.10) than in those without PCOS (12). Furthermore, PCOS symptoms in patients with both PCOS and obesity are significantly aggravated. However, the mechanism of PCOS has not been determined.

Adipose tissue dysfunction can lead to changes in adipokine levels (13). Many studies show that there are changes in adipokine levels in obese PCOS patients, indicating that adipokines may play a role in obese PCOS patients (14, 15). However, studies on adipokine levels in nonobese PCOS patients have been inconsistent. Moreover, it is not known whether the changes in adipokines are related directly to PCOS, to obesity, or to both. Thus, we aimed to perform this systematic review and meta-analysis to evaluate the levels of various adipokines in nonobese women with PCOS and in nonobese healthy controls.

## Materials and Methods

### Reporting Guidelines

This systematic review and meta-analysis was designed according to the Preferred Reporting Items for Systematic Reviews and Meta-Analyses (PRISMA) statement (16) and the Meta-analysis of Observational Studies in Epidemiology (MOOSE) guidelines (17).

### Eligibility Criteria

Studies published before July 2019 that define PCOS in line with the Rotterdam Criteria or other compatible criteria were considered for eligibility. The following studies were excluded: 1) studies on obese (BMI ≥30.0) women with PCOS; 2) reviews, nonhuman studies, and conference proceedings; 3) studies without control groups; and 4) studies without extractable data (data not provided as mean ± standard deviation [SD]).

### Databases

To identify eligible studies, an exhaustive literature search was performed in the PubMed, Embase, and Web of Science databases without restricting by location, journal, or language.

### Search Strategy

The search strategy involved the identification of studies on the levels of adiponectin, apelin, chemerin, irisin, leptin, omentin, resistin, vaspin, and/or visfatin, using the following keywords: (“polycystic ovary syndrome” OR “PCOS”) AND (“adiponectin” OR “apM-1 protein” OR “ACRP30 protein”) OR (“apelin”) OR (“chemerin” OR “TIG2 protein”) OR (“irisin” OR “FRCP2 protein”) OR (“leptin” OR “obese protein”) OR (“omentin” OR “intelectin 1”) OR (“resistin”) OR (“vaspin” OR “SERPINA12 protein”) OR (“visfatin” OR “NAMPT protein”).

### Study Selection and Data Extraction

Two reviewers independently reviewed the studies and determined whether they met the eligibility criteria. Disagreements between the two reviewers were resolved by discussion and, if necessary, consultation with a third reviewer. The following information was extracted: general study characteristics (name of the first author, year of publication, and study location), age and body mass index (BMI) of participants, summary of study conclusions, and data on adipokine levels.

### Assessment of Risk of Bias and Publication Bias

We used the Cochrane Collaboration tool to assess the risk of bias in the included studies. This tool involves seven domains: random sequence generation, allocation concealment, blinding of participants and personnel, outcome assessment, incomplete outcome data, selective reporting, and other biases. Each domain was independently evaluated (as having a high, low, or unclear risk of bias) by two reviewers, and disagreements were resolved by discussion. The possibility of publication bias was assessed by visual inspection of a funnel plot and Egger’s test.

### Statistical Analysis

Review Manager version 5.3 was used to perform the meta-analysis of the differences in mean adipokine levels between nonobese PCOS patients and nonobese healthy controls and to construct the forest plots. Due to the different measurement methods among studies, Review Manager was used to calculate the standardized mean difference (SMD) with 95% confidence intervals (CIs). Cochran’s Q test and the I^2^ test were used to assess the heterogeneity among studies. We selected random or fixed effects models according to whether high/moderate or low heterogeneity, respectively, was detected. STATA version 15.0 was used to conduct Egger’s test to assess the publication bias (with *P<*0.05 indicating publication bias), and the trim-and-fill method was used to recalculate the SMD if publication bias was detected.

## Results

### Study Selection

**Figure 1:**
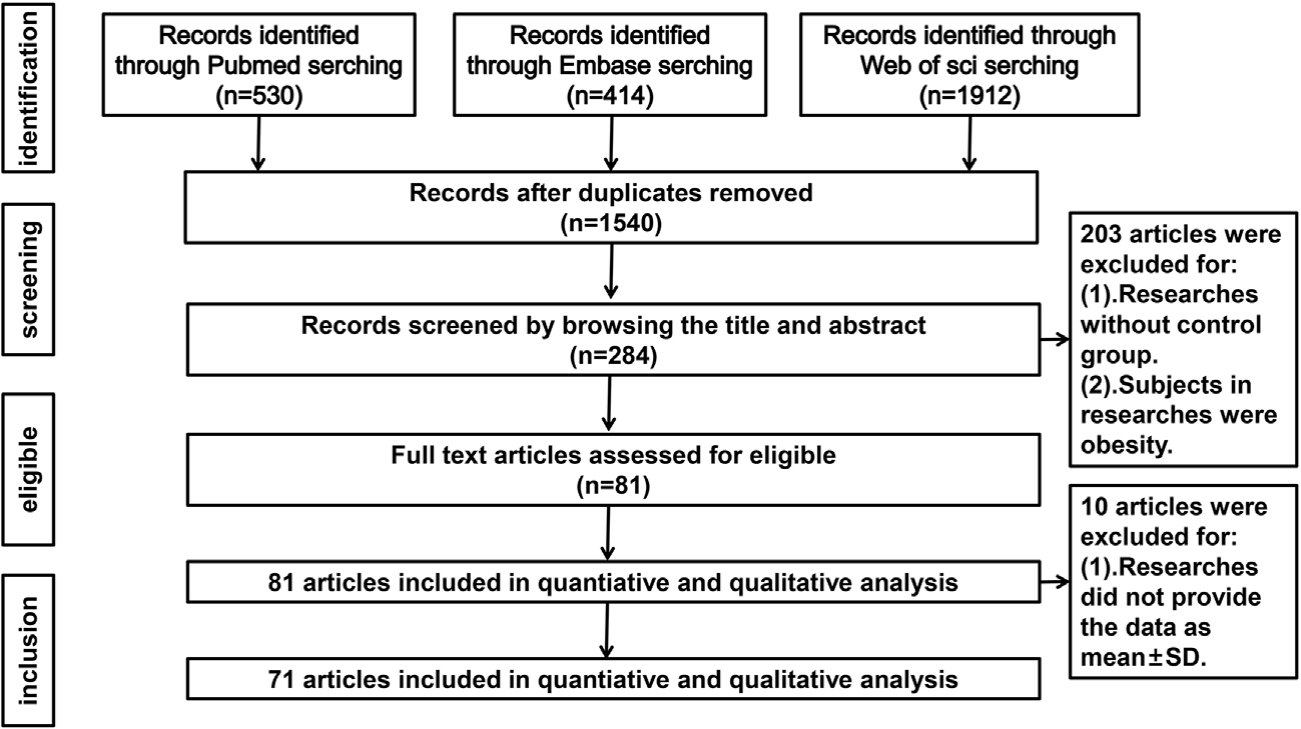
Flow chart of study inclusion in systematic review and meta-analysis.

Our search strategy identified 1540 articles, but 1256 were excluded after screening the titles and abstracts. Thereafter, 284 potentially relevant articles were assessed by reviewing the full text. Among these articles, 203 were excluded due to lack of a control group or inclusion of obese subjects and 10 were excluded because the data were not presented as mean ± SD. Thus, 71 studies (comprising 2495 PCOS patients and 2520 controls) met our eligibility criteria. **Figure 1** shows the flow diagram of study selection.

### Characteristics of Included Studies

The features of the included studies are presented in **Table 1**. Among the 81 included studies, several sets of PCOS diagnostic criteria were used (53 studies used the Rotterdam criteria, 9 used the National Institutes of Health (NIH) criteria; 3 used the ESHRE/ASRM consensus criteria, 2 used the National Institute of Child Health and Human Disease criteria, and the other studies used specific standards set out in the studies themselves). The studies were conducted in 26 countries (19 in Turkey, 8 in China, 8 in Poland, 7 in Greece, 4 in Egypt, 4 in Italy, 4 in Iran, 3 in India, 3 in South Korea, 3 in Taiwan, 2 in Brazil, 2 in Saudi Arabia, 1 in Croatia, 1 in the Netherlands, 1 in Pakistan, 1 in Denmark, 1 in France, 1 in the United States, 1 in Israel, 1 in Indonesia, 1 in Iraq, 1 in Germany, 1 in Japan, 1 in Australia, 1 in Spain, and 1 in Qatar). In the vast majority of the studies, the age of most participants was between 20 and 25 years. As there were no significant differences in age between the PCOS patients and the controls in any of the studies, we can exclude the influence of age on the results. The participants all had a BMI <30.0 (the World Health Organization criterion for obesity is BMI ≥30.0). **Table 1** also briefly lists the primary conclusions of each study.

**Table 1:** Characteristics of individual studies included in the systematic review and meta-analysis.

Study	PCOS diagnosis criteria	Region	Ethnicity	Age (PCOS vs. controls)	BMI (kg/m2)(PCOS vs. controls)	Primary conclusion

Ademoglu (2014)	Rotterdam Criteria	Turkey	NA	24.0 ± 4.8; 26.2 ± 4.9	21.8 ± 1.8; 21.3 ± 2.7	Serum chemerin increased in PCOS	
Akbarzadeh (2012)	Rotterdam Criteria	Iran	NA	21.68 ± 4.01; 24.06 ± 6.58	22.58 ± 2.14; 21.87 ± 1.83	Serum vaspin decreased in PCOS	
Ali (2016)	Rotterdam Criteria	Iraq	NA	NA; 20 to 40	25.074 ± 0.456; 25.022 ± 0.683	Serum irisin remained unchanged in PCOS	
Altinkaya (2014)	Rotterdam Criteria	Turkey	NA	23.4 ± 5.2; 24.1 ± 4.9	22.4 ± 1.5; 21.3 ± 1.2	Serum apelin decreased in PCOS	
Ardawi (2005)	NIH Criteria	Saudi Arabia	NA	25.9 ± 5.8; 26.55 ± 5.34	22.7 ± 2.4; 22.4 ± 1.8	Plasma adiponectin decreased in PCOS	
Arikan (2009)	Rotterdam Criteria	Turkey	NA	21.8 ± 5.4; 24.9 ± 5.7	23.8 ± 6.6; 23.1 ± 5.8	Serum adiponectin decreased and Serum leptin serum remained unchanged in PCOS
Baldani (2019)	Rotterdam Criteria	Croatia	NA	26.4 ± 5.9; 26.4 ± 2.7	22.4 ± 1.7; 21.8 ± 1.8	Serum adiponectin decreased and Serum leptin and resistin increased in PCOS
Bannigida (2018)	Rotterdam Criteria	India	Indian	18 to 40	25.6 ± 2.53; 21.2 ± 4.86	Serum adiponectin decreased and Serum visfatin increased in PCOS
Behboudi (2017)	NIH Criteria	Iran	NA	28.8 ± 5; 30.9 ± 6	21.8 ± 1.9; 22 ± 1.9	Serum visfatin decreased and Serum adiponectin, leptin, omentin, chemerin, and resistin increased in PCOS
Bousmpoula (2019)	Rotterdam Criteria	Greece	NA	31.9 ± 4.3; 35.1 ± 4.5	22.2 ± 1.1; 22.3 ± 1.2	Serum irisin increased in PCOS
Carmina (2005)	hyperan drogenism and chronic anovulation	American	NA	NA	22.7 ± 0.4; 23 ± 0.22	Serum adiponectin decreased and serum leptin and resistin increased in PCOS
Cassar (2014)	Rotterdam Criteria	Australia	NA	27 ± 4; 28 ± 6	23 ± 2; 22 ± 2	Serum visfatin remained unchanged in PCOS
Chen (2014)	Androgen Excess Society Criteria	Taiwan	Taiwan Chinese	25.1 ± 5.7; 28.5 ± 6.4	20.4 ± 1.7; 20.2 ± 2.1	Serum adiponectin increased and serum leptin and resistin decreased in PCOS
Cheng (2014)	Rotterdam Criteria	China	Chinese	28.3 ± 9.2; 28.6 ± 9.4	22.4 ± 2.5; 21.9 ± 2.6	Serum leptin increased in PCOS
Daan (2016)	Rotterdam Criteria	Netherlands	Caucasian	28.8 (25.8–31.2); 34.5 (30.7–37.7)	21.8 (19.8–22.2); 22.5 (21.2–24.5)	Serum adiponectin increased in PCOS
Daghestani (2018)	Rotterdam Criteria	Saudi Arabia	Saudi females	25.61 ± 0.39; 24.67 ± 0.50	22.86 ± 0.20; 20.85 ± 0. 24	Serum leptin remained unchanged in PCOS
Demirel (2007)	NIH Criteria	Turkey	Caucasian	15.5 ± 0.9; 15.5 ± 1.3	21.6 ± 2.4; 21.1 ± 2.1	Serum leptin increased in PCOS
Dikmen (2010)	Rotterdam Criteria	Turkey	NA	21.1 ± 2.5; 22.7 ± 2.2	22.3 ± 2.8; 20.6 ± 2.1	Serum visfatin increased in PCOS
Dikmen (2011)	Rotterdam Criteria	Turkey	NA	21.11 ± 2.59; 22.70 ± 2.25	22.36 ± 2.88; 20.64 ± 2.12	Serum resistin decreased in PCOS
Ducluzeau (2003)	NA	France	Caucasian	21 ± 3.3; 24.4 ± 4.5	22.1 ± 2.7; 22.9 ± 5.1	Plasma adiponectin decreased and plasma leptin remained unchanged in PCOS
Elorabi (1999)	NA	Egypt	NA	NA	NA	Serum leptin increased in PCOS
Erel (2003)	Specific standards in the article	Turkey	NA	22.0 ± 3.5; 28.0 ± 5.4	21.5 ± 2.2; 20.8 ± 2.0	Serum leptin remained unchanged in PCOS
Farshchian (2014)	NIH Criteria	Iran	NA	28.3 ± 5.1; 28.3 ± 4.8	22.5 ± 1.7; 22.4 ± 1.8	Serum resistin and visfatin remained unchanged in PCOS
Foda (2018)	Rotterdam 2004	Egypt	NA	28.25 ± 2.08; 28.6 ± 2.11	23.38 ± 0.34; 23.88 ± 0.45	Serum irisin increased in PCOS
Foda et al. (18)	Rotterdam Criteria	Egypt	Egyptian	21 to 26; NA	24.64 ± 0.36; 23.914 ± 0.55	Serum chemerin increased in PCOS
Foda et al. (18)	Rotterdam Criteria	Egypt	Egyptian	NA	NA	Serum irisin increased in PCOS
Garruti (2014)	ESHRE/ASRM consensus	Italy	Caucasian	32.09 ± 3.60; 34.35 ± 3.13	22.55 ± 2.61; 22.55 ± 2.55	Serum leptin increased in PCOS
Gen (2009)	Rotterdam Criteria	Turkey	NA	21.85 ± 4.06; 23.46 ± 5.15	20.74 ± 1.75; 20.85 ± 2.08	Serum visfatin remained unchanged in PCOS
Güdücü (2012)	Rotterdam Criteria	Turkey	NA	24.03 ± 4.08; 27.70 ± 5.06	21.03 ± 1.94; 23.45 ± 5.32	Serum visfatin remained unchanged in PCOS
Gul (2015)	Rotterdam Criteria	Turkey	NA	23.7 ± 3.1; 29.8 ± 4.1	22.5 ± 2.0; 24.2 ± 2.7	Plasma visfatin and resistinin remained unchanged in PCOS
Gümüş (2014)	Rotterdam Criteria	Turkey	NA	18.80 ± 2.20; 19.61 ± 2.41	24.06 ± 5.22; 21.30 ± 3.89	Serum visfatin remained unchanged in PCOS
Güne (2015)	Androgen Excess Society Criteria	Turkey	NA	NA	22.65 ± 2.05; 21.41 ± 3.44	Serum omentin decreased in PCOS
Guven (2009)	Rotterdam Criteria	Turkey	NA	15.7 ± 1; 15.1 ± 0.8	20.1 ± 1; 20.1 ± 1	Serum leptin and adiponectin remained unchanged in PCOS
Guvenc (2016)	Rotterdam Criteria	Turkey	NA	25.40 ± 5.62; 31.50 ± 7.59	24.87 ± 5.02; 23.7 ± 4.46	Serum vaspin, chemerin, and omentin remained unchanged in PCOS
Hahn (2006)	NIH Criteria	Germany	NA	27.0 ± 5.6; 25.0 ± 4.0	21.0 ± 1.9; 21.5 ± 2.0	Serum leptin decreased in PCOS
Jeon (2013)	Rotterdam Criteria	Korea	Korean	23.88 ± 4.86; 24.92 ± 2.94	20.23 ± 2.19; 19.77 ± 1.51	Serum leptin increased in PCOS
Kim (2006)	Rotterdam Criteria	Korea	Korean	26.3 ± 6.1; 25.5 ± 5.3	21.3 ± 1.8; 21.7 ± 1.4	Serum adiponectin remained unchanged in PCOS
Koiou (2011)	Rotterdam Criteria	Greece	NA	19.9 ± 3.0; 31.3 ± 4.5	23.2 ± 4.4; 21.9 ± 1.6	Serum vaspin increased in PCOS
Korczala (2008)	ESHRE/ASRM consensus	Poland	NA	22 ± 2.5; 21 ± 2.3	21 ± 0.9; 22 ± 1.3	Serum resistin remained unchanged in PCOS
Kowalska (2007)	Rotterdam Criteria	Poland	NA	23.69 ± 3.46; 26.24 ± 6.00	21.39 ± 2.10; 21.81 ± 2.00	Serum visfatin increased in PCOS
Kruszynska (2014)	Rotterdam Criteria	Poland	NA	16 to 40; 17 to 40	21.03 ± 1.83; 20.76 ± 2.09	Plasma and serum adiponectin decreased in PCOS
Lecke (2011)	Rotterdam Criteria	Brazil	NA	25.4 ± 5.3; 29.3 ± 5.9	22.5 ± 2.3; 22.5 ± 1.9	Serum leptin increased in PCOS
Lee (2013)	ESHRE/ASRM consensus	Korea	Korean	24 ± 5; 24 ± 4	21.9 ± 2.0; 21.9 ± 2.0	Plasma and serum adiponectin decreased in PCOS
Li (2009)	Rotterdam Criteria	China	Chinese	25±; 26 ± 7	20.3 ± 1.9; 20.6 ± 2.1	Serum leptin and adiponectin remained unchanged in PCOS
Lu (2005)	Specific standards in the article	China	Chinese	30 (29.0-32.5); 30 (29.0-32.8)	21.6 ± 2.0; 21.4 ± 2.2	Serum leptin increased in PCOS
Macut (1997)	Specific standards in the article	Spain	Caucasian	24.3 ± 1.56; 28.0 ± 1.25	21.9 ± 0.71; 21.01 ± 0.58	Serum leptin remained unchanged in PCOS
Mancini (2009)	Specific standards in the article	Italy	caucasian (native)	18 to 35; NA	NA	Serum leptin decreased in PCOS
Mendonca (2004)	NIH Criteria	Brazil	NA	20.0 ± 5.2; 30.0 ± 6.3	23.2 ± 2.3; 22.3 ± 2.2	Serum leptin remained unchanged in PCOS
Mirza (2014)	Rotterdam Criteria	Pakistan	NA	25.7±; 25.4 ± 6.7	19.3 ± 2.6; 18.3 ± 2.4	Serum adiponectin decreased in PCOS
Nambiar et al. (19)	Rotterdam Criteria	India	Indian	28.71 ± 5.49; 29.88 ± 4.69	22.15 ± 1.64; 21.83 ± 1.772	Serum adiponectin decreased and serum resistin increased in PCOS
Olszanecka (2011)	Rotterdam Criteria	Poland	NA	24.0 ± 6.9; 27.8 ± 7.1	22.1 ± 2.4; 22.1 ± 2.1	Plasma adiponectin decreased and plasma resistin remained unchanged in PCOS
Olszanecka (2012)	Rotterdam Criteria	Poland	NA	23.7 ± 4.5; 23.8 ± 4.3	21.3 ± 2.2; 22.2 ± 2.0	Plasma visfatin decreased in PCOS
Olszanecka (2013)	Rotterdam Criteria	Poland	NA	23.7 ± 4.5; 23.8 ± 4.3	21.3 ± 2.2; 22.2 ± 2.0	Plasma adiponectin remained unchanged in PCOS
olszanecka (2015)	Rotterdam Criteria	Poland	NA	23.7 ± 4.5; 23.8 ± 4.3	21.3 ± 2.2; 22.2 ± 2.0	Plasma apelin increased in PCOS
Orio (2003)	Rotterdam Criteria	Italy	Caucasian	29.6 ± 1.1; 29.8 ± 1.1	22.1 ± 0.3; 22.0 ± 0.4	Plasma adiponectin remained unchanged in PCOS
Orio (2004)	NIH Criteria	Italy	Caucasian	NA	NA	Plasma adiponectin remained unchanged in PCOS
Orlik (2014)	Rotterdam Criteria	Poland	NA	23.7 ± 4.5; 23.8 ± 4.3	20.6 (19.6-22.7); 22.4 (21.0-24.0)	Serum adiponectin decreased in PCOS
Pangaribuan (2011)	Rotterdam Criteria	Indonesia	NA	25.6 ± 6.1; 22.2 ± 2.1	22.0 ± 1.7; 20.6 ± 2.1	Serum resistin remained unchanged in PCOS
Panidis (2003)	Specific standards in the article	Greece	NA	25.7 ± 4.0; 27.8 ± 4.9	21.6 ± 1.6; 20.5 ± 2.0	Serum adiponectin decreased in PCOS
Panidis (2008)	NIH Criteria	Greece	NA	23.78 ± 0.95; 24.31 ± 0.90	22.09 ± 0.41; 21.62 ± 0.38	Serum visfatin increased in PCOS
Panidis (2004)	Specific standards in the article	Greece	NA	25.7 ± 4.0; 28.6 ± 4.5	21.6 ± 1.6; 21.6 ± 1.9	Serum resistin remained unchanged in PCOS
Pekcan (2019)	Rotterdam Criteria	Turkey	NA	21.81 ± 3.78; 22.97 ± 4.16	22.10 ± 2.91; 21.58 ± 2.81	Serum adiponectin decreased in PCOS
pinhas (2009)	Rotterdam Criteria	Israel	NA	15.5 ± 1.7; 14.2 ± 2.1	20.1 ± 2; 18.6 ± 1.7	Serum adiponectin decreased in PCOS
Plati (2010)	Rotterdam Criteria	Greece	NA	32.3 ± 4.0; 32.9 ± 4.3	22.41 ± 0.21; 22.5 ± 0.2	Serum visfatin decreased in PCOS
Ram (2005)	Specific standards in the article	India	NA	18 to 44	20.0 ± 1.42; 19.0 ± 1.02	Serum leptin increased in PCOS
Rizk (2015)	NIH Criteria	Qatar	NA	21 ± 1.3; 21.1 ± 1.85	20.867 ± 2.41; 20.93 ± 3.03	Serum leptin increased in PCOS
Seow (2007)	Rotterdam Criteria	Taiwan	Taiwan Chinese	32.2 ± 3.5; 28.3 ± 3.3	21.1 ± 1.7; 22.5 ± 14.9	Serum resistin remained unchanged in PCOS
Seow (2009)	Rotterdam Criteria	Taiwan	Taiwan Chinese	30.1 ± 4.4; 27.6 ± 3.3	21.3 ± 5.83; 22.8 ± 5.6	Serum adiponectin decreased in PCOS
Sharifi (2010)	Rotterdam Criteria	Iran	Aryan	NA	NA	Serum adiponectin decreased in PCOS
Sun (2015)	Rotterdam Criteria	China	Chinese	27.45 ± 3.61; 26.70 ± 4.40	21.80 ± 1.89; 20.39 ± 2.17	Serum apelin increased in PCOS
Svendsen (2012)	Rotterdam Criteria	Denmark	NA	NA	NA	Plasma adiponectin decreased in PCOS
Takeuchi (2000)	Specific standards in the article	Japan	NA	26.3 ± 1.3; 26.9 ± 1.6	19.4 ± 0.5; 19.0 ± 0.5	Serum leptin remained unchanged in PCOS
Telli (2002)	Rotterdam Criteria	Turkey	Caucasian	21.4 ± 2.74; 22.71 ± 5.44	23.9 ± 4.91; 22.06 ± 3.98	Serum leptin in increased in PCOS
Tsouma (2014)	Rotterdam Criteria	Greece	NA	31.0 (24.0–41.0); 31.6 (26.0–42.0)	22.2 (20.4–24.5); 22.5 (20.0–24.5)	Serum visfatin increased in PCOS
Wang (2010)	Specific standards in the article	China	Chinese	17 to 3; NA	NA	Serum adiponectin decreased and serum resistin increased in PCOS
Wang (2012)	Rotterdam Criteria	China	Chinese	25.7 ± 4.5; 26.8 ± 4.7	21.6 ± 2.6; 21.7 ± 1.9	Serum resistin remained unchanged in PCOS
Xiao (2006)	Obstetrics and gynecology, 6th edition	China	Chinese	27.7 ± 3.56; 28.35 ± 2.98	21.63 ± 2,8; 20.51 ± 1.45	Serum leptin increased in PCOS
Yang (2015)	Rotterdam Criteria	China	Chinese	24.62 ± 4.41; 3.73 ± 3.39	20.86 ± 2.16; 20.10 ± 1.26	Serum chemerin increased in PCOS
Yasar (2011)	Rotterdam Criteria	Turkey	NA	17.45 ± 0.99; 17.19 ± 0.40	21.43 ± 2.07; 18.64 ± 1.58	Serum adiponectin decreased in PCOS
Yildizhan (2011)	Rotterdam Criteria	Turkey	NA	25.70 ± 3.70; 25.44 ± 2.62	23.85 ± 1.1; 23.88 ± 3.83	Serum leptin increased in PCOS
Yilmaz (2009)	Rotterdam Criteria	Turkey	NA	NA	21.43 ± 3.12; 20.89 ± 3.21	Serum adiponectin decreased and serum resistin increased in PCOS

PCOS, polycystic ovary syndrome; BMI, body mass index; NA, not available; NIH, National Institutes of Health.

### Associations of PCOS With Adipokine Levels

**Figure 2:**
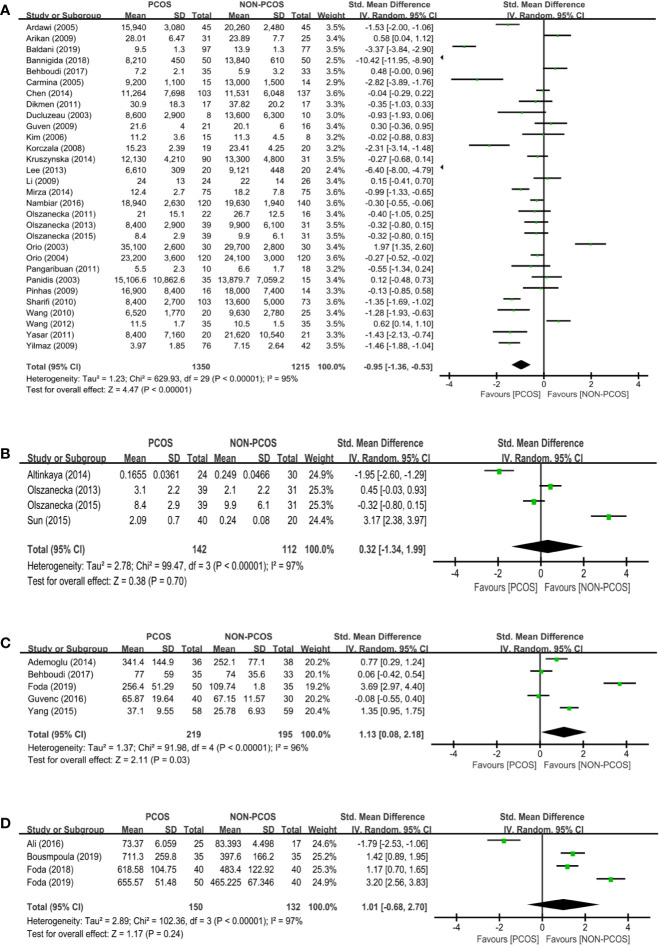
Meta-analysis of the association between the levels of adipokines in nonobese patients and PCOS. Weights are from random effects analysis. **(A)** Meta-analysis of adiponectin. **(B)** Meta-analysis of apelin. **(C)** Meta-analysis of chemerin. **(D)** Meta-analysis of irisin. PCOS, polycystic ovary syndrome; CI, confidence interval; SD, standard difference.

Thirty studies (*n*=2565 participants) compared the adiponectin level between nonobese PCOS patients and controls (**Figure 2A**), and there was significant heterogeneity among the studies (*I^2^ = *95%; *P*<0.00001). PCOS was significantly associated with a decreased adiponectin level (SMD: -0.95; 95% CI: -1.36 to -0.53; *P*<0.00001).

Four studies (*n*=254 participants) compared the apelin level between nonobese PCOS patients and controls (**Figure 2B**). There was no significant association between PCOS and apelin (SMD: 0.32; 95% CI: -1.34 to 1.99; *P*=0.70).

Five studies (*n*=414 participants) compared the chemerin level between nonobese PCOS patients and controls (**Figure 2C**), and there was significant heterogeneity among the studies (*I* ^2^ = 96%; *P*<0.00001). PCOS was significantly associated with an increased chemerin level (SMD: 1.13; 95% CI: 0.08 to 2.18; *P*=0.03).

Four studies (*n*=282 participants) compared the irisin level between nonobese PCOS patients and controls (**Figure 2D**), and there was significant heterogeneity among the studies (*I^2^ = *97%; *P*<0.00001). There was no significant association between PCOS and irisin (SMD: 1.01; 95% CI: -0.68 to 2.70; *P*=0.24).

**Figure 3:**
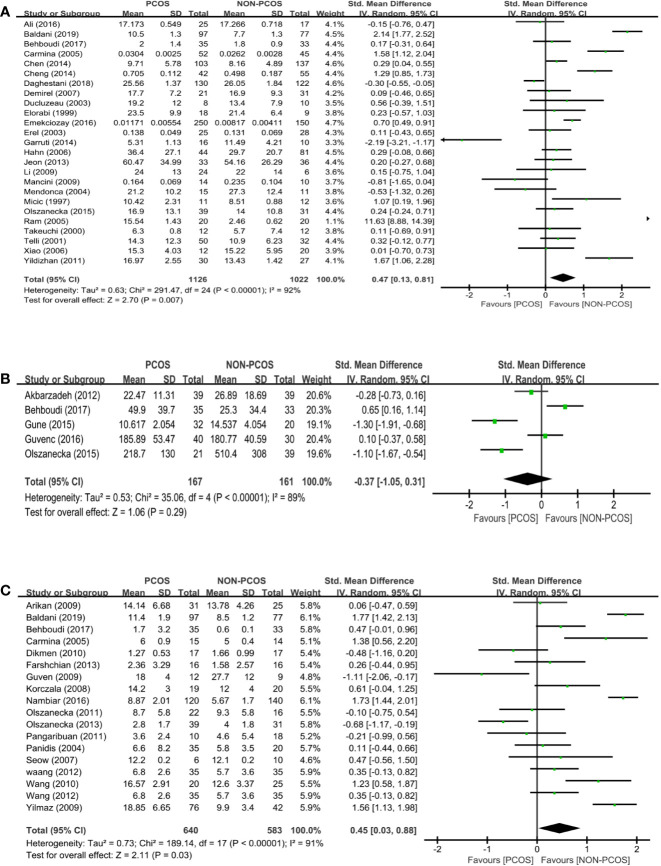
Meta-analysis of the association between the levels of adipokines in nonobese patients and PCOS. Weights are from random effects analysis. **(A)** Meta-analysis of leptin. **(B)** Meta-analysis of omentin. **(C)** Meta-analysis of resistin. PCOS, polycystic ovary syndrome; CI, confidence interval; SD, standard difference.

Twenty-five studies (*n*=2148 participants) compared the leptin level between nonobese PCOS patients and controls (**Figure 3A**). PCOS was significantly associated with an increased leptin level (SMD: 0.47; 95% CI: 0.13 to 0.81; *P*=0.007).

Five studies (*n*=328 participants) compared the omentin level between nonobese PCOS patients and controls (**Figure 3B**), and there was significant heterogeneity among the studies (*I^2^ = *89%; *P*<0.00001). There was no significant association between PCOS and omentin (SMD: -0.37; 95% CI: -1.05 to 0.31; *P*=0.29).

Eighteen studies (*n*=1223 participants) compared the resistin level between nonobese PCOS patients and controls (**Figure 3C**), and there was significant heterogeneity among the studies (*I^2^ = *91%; *P*<0.00001). PCOS was associated with an increased resistin level (SMD: 0.45; 95% CI: 0.03 to 0.88; *P*=0.03; *n*=18 studies).

**Figure 4:**
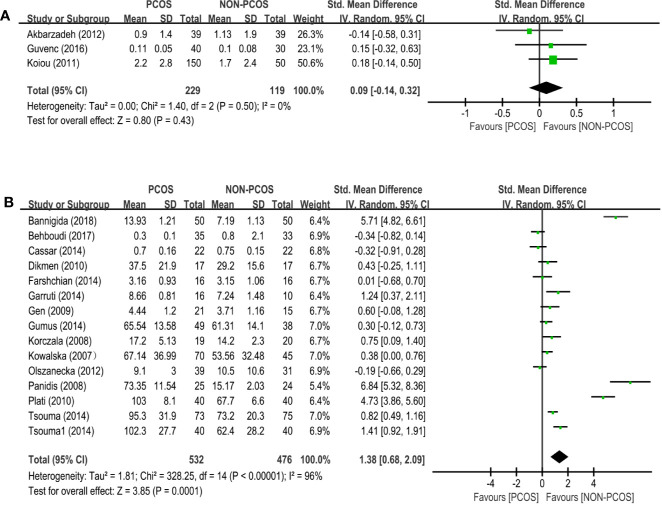
Meta-analysis of the association between the levels of adipokines in nonobese patients and PCOS. Weights are from random effects analysis. **(A)** Meta-analysis of vaspin. **(B)** Meta-analysis of visfatin. PCOS, polycystic ovary syndrome; CI, confidence interval; SD, standard difference.

Three studies (*n*=348 participants) compared the vaspin level between nonobese PCOS patients and controls (**Figure 4A**). There was no significant association between PCOS and vaspin (SMD: 0.09; 95% CI: -0.14 to 0.32; *P*=0.43).

Twenty-six studies (*n*=1008 participants) compared the visfatin level between nonobese PCOS patients and controls (**Figure 4B**), and there was significant heterogeneity among the studies (*I* ^2^ = 96%; *P*<0.00001). PCOS was associated with an increased visfatin level (SMD: 1.38; 95% CI: 0.68 to 2.09; *P*=0.0001).

### Publication Bias

**Figure 5:**
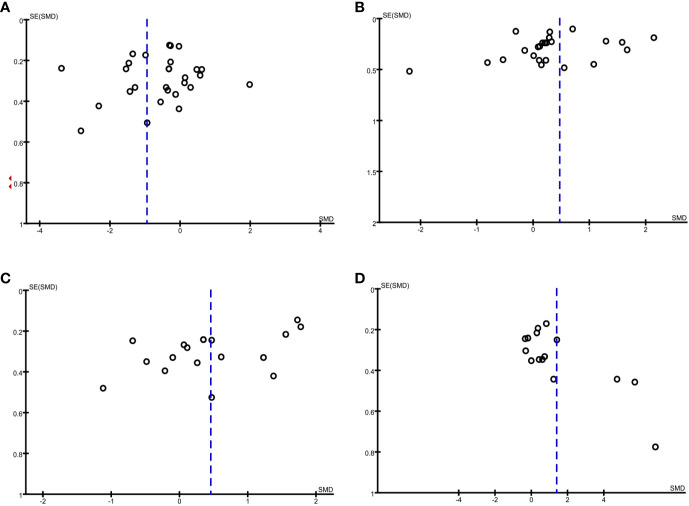
Funnel plot analysis to detect publication bias under a dominant model. **(A)** Funnel plot for adiponectin. **(B)** Funnel plot for leptin. **(C)** Funnel plot for resistin. **(D)** Funnel plot for visfatin.

The funnel plot method was used to detect any publication bias, and Egger’s test was used to quantify the publication bias. Publication bias can be due to systematic reviewers who, by not including unpublished literature, lead to final statistical conclusions that differ from actual results. Regarding adiponectin and resistin, the shape of the funnel plots was not obviously asymmetric. However, regarding leptin and visfatin, the funnel plots indicated observable publication bias (**Figures 5A–D**). Egger’s test shows no evidence of publication bias regarding adiponectin and leptin, but there is possible publication bias regarding resistin and visfatin (**Table 2**). We used the trim-and-fill method and recalculated our pooled SMD values for resistin and visfatin, and the corrected and uncorrected SMD values did not differ, which suggested that publication bias did not affect the final results. We did not assess the publication bias for apelin, chemerin, irisin, omentin, or vaspin, as the Cochrane Handbook for Systematic Reviews of Interventions (www.cochranehandbook.org) states that the test for publication bias yields unreliable results when <10 studies are included.

**Table 2:** Egger publication bias test for the adiponectin, leptin, visfatin, and resistin.

Indices	No. of studies	Coefficient	Standard Error	*t*	*P*	95% CI
adiponectin	30	-3.45	1.98	-1.75	0.092	-7.50 to 0.60
leptin	25	0.45	1.54	0.29	0.775	-2.75 to 3.64
visfatin	15	8.39	3.11	2.7	0.018	1.67 to 15.11
resistin	18	-6.48	1.95	-3.33	0.004	-10.61 to -2.35

CI, confidence interval.

## Discussion

To our knowledge, this is the first systematic review and meta-analysis quantifying the levels of nine adipokines in nonobese PCOS patients. This systematic review and meta-analysis of 81 studies demonstrates that nonobese PCOS patients have a significantly decreased adiponectin level and significantly increased leptin, visfatin, chemerin, and resistin levels. Thus, our results indicate that the concentration of common adipokines in plasma or serum were remarkably altered in nonobese PCOS patients.

Over the past decade, many studies have investigated circulating adipokine levels in nonobese PCOS patients. The results of the studies are inconsistent, with adipokine levels differing by geographical region, ethnicity, and age. For example, a study by Alfaqih et al. shows that adipokine levels in PCOS patients were significantly lower than those in controls (20), whereas a study by Arikan et al. shows significantly higher levels (21). The purpose of this meta-analysis was to integrate these results in a statistical analysis. Combining study findings is an attractive option to enhance the credibility of the evidence (22). Therefore, we performed a meta-analysis to reach a reliable conclusion on the changes in adipokine levels in nonobese PCOS patients.

Changes in adipokine levels in PCOS patients might be caused by obesity rather than the PCOS itself. This should be considered because adipokine levels are related to BMI (23), and PCOS patients tend to have a higher BMI (24) with a meta-analysis reporting higher rates of overweight (RR: 1.95; 95% CI: 1.52 to 2.50), obesity (RR: 2.77; 95% CI: 1.88 to 4.10), and central obesity (RR: 1.73; 95% CI: 1.31 to 2.30) in women with PCOS compared with women without PCOS (25). To exclude the influence of obesity and directly analyze the relationships between PCOS and adipokine levels, we only included studies on nonobese PCOS patients in our meta-analysis.

Understanding the dysregulation of adipokine levels in nonobese PCOS patients may help to explain the pathophysiology and symptoms of PCOS, which are thought to involve hyperandrogenism, IR, and chronic inflammation. 1) Hyperandrogenism: the occurrence of PCOS is closely related to hyperandrogenism (26). After androgen receptor knockdown in adipocytes in mice, the adipokine levels were altered, and the impaired insulin sensitivity and poor glucose tolerance were found not to be associated with obesity. Therefore, androgen receptors in adipocytes may influence adipokine levels, and they may control insulin sensitivity and glucose tolerance independently of obesity (27). Additionally, in nonobese PCOS patients, androgen hypersecretion and androgen receptor dysfunction may underlie the changes in the adipokine levels as they are the main signs of the condition (28). Moreover, sex hormones influence adipokine levels, which provides further evidence that the increase in androgens in PCOS patients might influence the adipokine levels (29). Furthermore, a study involving triptorelin treatment provided evidence that hyperandrogenemia may increase the level of the adipokine leptin (in addition to leptin influencing androgens). More precisely, although leptin may influence the gonadal axis, the gonadal axis itself may also dramatically upregulate leptin secretion as indicated by the significant associations in women between the percentage increase in circulating leptin levels (relative to baseline) and the percentage increases in both the free androgen index and the testosterone levels following triptorelin treatment (30). 2) IR: the occurrence of PCOS is closely related to IR (31). In vitro research has shown that, in fat cells, certain adipokines (such as omentin) activate protein kinases that increase insulin-mediated glucose transport, thus increasing insulin sensitivity (32). Additionally, high leptin levels inhibit proinsulin mRNA expression, involving decreased transcription activity of the insulin gene promoter and decreased phosphorylation of insulin receptors in peripheral tissues (33, 34). High leptin levels may lead to IR in nonobese PCOS patients by promoting the following events: (i) inhibition of phosphoenolpyruvate carboxykinase, causing decreased hepatic glucose oxidation and increased hepatic glycogen reserve; (ii) fat deposition in skeletal muscle cells; (iii) fat breakdown and free fatty acid production; and (iv) direct inhibition of basal insulin secretion and glucose-stimulated insulin secretion. Furthermore, the insulin sensitizer thiazolidinedione upregulates adiponectin mRNA expression and promotes the differentiation and apoptosis of adipocytes, thus reducing IR. Therefore, dysregulated adipokine levels may play important roles in IR in nonobese PCOS patients, and PCOS treatment could involve correcting the dysregulated adipokine levels. In particular, we need to consider the role of low serum adiponectin levels in causing IR in women with PCOS as adiponectin (i) decreases muscle triglyceride content, (ii) regulates the insulin receptor, (iii) activates peroxisome proliferator-activated receptor α (PPARα) to increase fatty acid oxidation, (iv) reduces hepatic glucose production by activating protein kinases, (v) increases fat oxidation in muscle, and (vi) inhibits the expression of gluconeogenic enzymes (35). 3) Chronic inflammation: The proinflammatory adipokine chemerin was discovered to promote the chemotaxis of immature dendritic cells and macrophages *via* its receptor CMKLR1 (36). Chemerin and CMKLR1 (which is expressed on immature dendritic cells, myeloid dendritic cells, macrophages, and natural killer cells) were later shown to recruit immune cells to injury sites and might affect the occurrence and development of inflammation (37, 38). Adipokines and their receptors are elevated in many inflammatory states, and adipokine levels might be predictors of PCOS. Additionally, obesity involves a chronic low-grade inflammatory state, and this long-term inflammatory stimulation may partly underlie the downregulation of omentin (which is an adipokine with anti-inflammatory, anticardiovascular disease, and antidiabetic effects) (39, 40).

Research shows that both environmental and genetic factors play important roles in the development of PCOS. Studies on whether insulin receptor gene mutations play a role in the pathogenesis of PCOS all yield negative results, indicating that these mutations are unlikely to be the main cause of IR in PCOS. However, CYP11A promoter polymorphisms are shown to be associated with the pathogenesis of PCOS. The CYP11A protein catalyzes the rate-limiting step of the pathway that produces pregnenolone (an androgen precursor) from cholesterol, so CYP11A promoter polymorphisms that upregulate its expression may upregulate ovarian or adrenal androgen secretion. However, the specific mechanism remains unclear, and there is conflicting experimental evidence regarding the fact that the polymorphisms cause increased androgen secretion (41).

In addition, the roles of adipokine gene polymorphisms in the pathogenesis of PCOS may be important. A Finnish study suggests that adiponectin gene polymorphisms may increase susceptibility to PCOS as the T allele studied is significantly reduced in PCOS patients compared to controls (odds ratio: 0.72; 95% CI: 0.52 to 0.99; *P*=0.047) (42). However, the adiponectin gene polymorphisms 45 T→G and 276 G→T are not significantly different between PCOS patients and controls. Nevertheless, these polymorphisms are associated with adiponectin levels and the levels of IR-associated metabolic variables. These polymorphisms may be in linkage disequilibrium with several other loci that primarily function to produce and polymerize adiponectin, thereby affecting adipokine levels (43). A follow-up study reports similar results, demonstrating that PCOS is not associated with the adiponectin 45 T→G and 276 G→T polymorphisms. In addition, hyperandrogenemia and abdominal obesity are the main causes of low serum adiponectin levels in PCOS patients, which may explain the link between the former two factors and the IR experienced by PCOS patients (as decreased adiponectin may cause IR) (44). Mice with a mutant leptin receptor (Y123F mice with three tyrosine residues [Tyr985, Tyr1077, and Tyr1138] replaced with phenylalanine) had atrophied ovaries and abnormal follicle development along with fewer ovarian leptin receptors, which may involve effects on JAK-STAT signaling and on hormone biosynthetic pathways (45). A study of PCOS patients and their parents reports that the resistin gene is near the D19S884 polymorphic marker, which is closely linked to PCOS, and both the resistin gene and D19S884 are reasonable candidate susceptibility loci for PCOS (46). A study of women in India shows that the resistin gene 420 C→G (promoter region) and 299 G→A polymorphisms are positively associated with the incidence of PCOS (with the heterozygous CG genotype, regarding the former polymorphism, being more common in PCOS patients than controls) (19). Both polymorphisms are significantly associated with cerebrovascular disease in type 2 diabetes patients and with the development of IR, which may explain the development of IR in PCOS patients (47). Another study shows that vaspin gene rs2236242 polymorphism is significantly different between PCOS patients and controls (odds ratio: 0.59; 95% CI: 0.37 to 0.95; *P*=0.03). However, after adjusting for BMI, the results are not significant (48). Overall, studies on the role of adipokine gene polymorphisms in PCOS are still relatively rare and inconsistent, so more research is needed to demonstrate their roles.

Nonobese PCOS patients with dysregulated adipokine levels can develop related diseases over time, such as type 2 diabetes, coronary heart disease, and hypertension (49). Type 2 diabetes is almost 10 times more common in PCOS patients than in the normal population, and glucose intolerance is 30%–50% higher in obese PCOS patients (50). The risk of cardiovascular disease in PCOS patients should also not be ignored. As PCOS is closely related to IR, this can lead to a variety of abnormalities, such as hypertension, glucose intolerance, diabetes, metabolic syndrome, and dyslipidemia, which increase the risk of cardiovascular disease in women (51).

Adipokine levels may be of great significance in the diagnosis and treatment of PCOS, and increased understanding of the abnormal changes in adipokine levels is required. Metformin might alleviate the symptoms and treat PCOS by correcting the abnormal levels of certain adipokines, such as resistin, visfatin, irisin, and chemerin levels (18, 52–54). For example, metformin may alleviate abnormal visfatin levels in PCOS patients by increasing peripheral insulin sensitivity and thereby decreasing the IR-induced hyperinsulinemia, which, in turn, may lower visfatin secretion because insulin and glucose affect visfatin secretion *via* the phosphatidylinositol 3-kinase/protein kinase B pathway (55). It is believed that adipokines and hyperandrogenemia form a vicious endocrine metabolism cycle, increasing the risk of PCOS and other related endocrine diseases. Relatedly, metformin may also alleviate PCOS symptoms *via* reducing high testosterone levels (56). However, more studies are needed to prove whether metformin can correct dysregulated adipokine levels and, thus, treat PCOS.

There is significant statistical heterogeneity among the studies that assess adiponectin, apelin, chemerin, irisin, leptin, omentin, resistin, and visfatin levels. This might reflect clinical heterogeneity and, more specifically, differences in the PCOS diagnostic criteria used. Differences in location, ethnicity, age, and/or BMI may also have played a role. For example, the BMI thresholds used to determine obesity differ among the studies. Caution should be exercised when extrapolating the results to other situations. However, the analysis of apelin shows no heterogeneity, indicating that the included studies are homogeneous and the study results are accurate.

Furthermore, we find evidence of publication bias. Publication bias is a phenomenon in the medical literature that involves positive findings having a greater chance of being published. Performing a meta-analysis after using the trim-and-fill method to correct for funnel plot asymmetry arising from publication bias can indicate whether publication bias has impacted the results. Our results for resistin and visfatin after using the trim-and-fill method suggest that publication bias did not affect the conclusions (i.e., PCOS remained significantly associated with increased resistin and visfatin levels). However, some studies came to a different conclusion (i.e., PCOS is not associated with resistin or visfatin levels), so more consideration is still needed before potentially applying the results in clinical practice.

Our literature search was comprehensive, and we did not apply any region, journal, or language restrictions that would limit the assessment of the relationships between the adipokine levels and PCOS. However, our meta-analysis still has several limitations. First, many different sets of criteria are used for the identification of PCOS, so the diagnosis of PCOS is associated with heterogeneity. Second, different regions have different definitions of obesity; for example, the World Health Organization developed the world standard of BMI >30 for obesity although BMI >28 is considered to be obese in China. Given this, not all obese PCOS patients (e.g., based on the less stringent criteria of BMI >28) may have been excluded, so obesity may have influenced the results. Third, many different methods are used to determine the adipokine levels in the blood, which may have led to some deviations among the studies. Last, many studies demonstrate that PCOS patients are much more likely to develop hyperandrogenemia, and we did not rule out the effect of androgen levels on adipokine levels. Despite these limitations, our meta-analysis increased the statistical power by pooling the results of single studies, which meant that the total number of subjects was sufficiently large to support more robust conclusions.

In summary, this systematic review and meta-analysis demonstrates that the circulating adipokine levels in nonobese PCOS patients are changed to varying degrees relative to those in nonobese healthy controls. This may help to identify the pathogenesis of PCOS and new biochemical diagnostic criteria for PCOS. Moreover, these results might provide insights into new PCOS treatment approaches involving correcting dysregulated adipokine levels. However, further studies are needed to explore the potential mechanisms underlying the dysregulation in adipokine levels and to determine whether the PCOS could be treated by correcting this dysregulation.

## Author Contributions

KL conducted the literature search, compiled the data, and drafted the manuscript. XS and XW contributed to the literature search and data interpretation. HW reviewed the manuscript and provided advice. XC contributed to critical discussion, provided extensive advice, and reviewed and revised the manuscript drafts. All authors contributed to the article and approved the submitted version.

## Funding

This work was supported by Wenzhou Municipal Science and Technology Bureau (Y20180271) and the Health and Family Planning Commission of Zhejiang Province (2019317125).

## Conflict of Interest

The authors declare that the research was conducted in the absence of any commercial or financial relationships that could be construed as a potential conflict of interest.
